# Peripheral CD8+CD28+ T lymphocytes predict the efficacy and safety of PD-1/PD-L1 inhibitors in cancer patients

**DOI:** 10.3389/fimmu.2023.1125876

**Published:** 2023-03-10

**Authors:** Ruixuan Geng, Hui Tang, Tingting You, Xiuxiu Xu, Sijian Li, Zepeng Li, Yuan Liu, Wei Qiu, Na Zhou, Ningning Li, Yuping Ge, Fuping Guo, Yuhong Sun, Yingyi Wang, Taisheng Li, Chunmei Bai

**Affiliations:** ^1^ Department of International Medical Services, Peking Union Medical College Hospital, Chinese Academy of Medical Sciences and Peking Union Medical College, Beijing, China; ^2^ Department of Medical Oncology, Peking Union Medical College Hospital, Chinese Academy of Medical Sciences and Peking Union Medical College, Beijing, China; ^3^ Department of Obstetrics and Gynecology, Peking Union Medical College Hospital, Chinese Academy of Medical Sciences and Peking Union Medical College, National Clinical Research Center for Obstetric and Gynecologic Diseases, Beijing, China; ^4^ Department of Laboratory Medicine, Peking Union Medical College Hospital, Chinese Academy of Medical Sciences and Peking Union Medical College, Beijing, China; ^5^ Department of Infectious Diseases, Peking Union Medical College Hospital, Chinese Academy of Medical Sciences and Peking Union Medical College, Beijing, China; ^6^ Department of Radiation Oncology, Dandong First Hospital, Dandong, Liaoning, China

**Keywords:** programmed cell death-1, lymphocyte subsets, CD8+CD28+ T cell, prognosis, immune-related adverse events

## Abstract

**Background:**

Programmed cell death protein-1/programmed cell death ligand-1 (PD-1/PD-L1) inhibitors works by reactivating immune cells. Considering the accessibility of noninvasive liquid biopsies, it is advisable to employ peripheral blood lymphocyte subsets to predict immunotherapy outcomes.

**Methods:**

We retrospectively enrolled 87 patients with available baseline circulating lymphocyte subset data who received first-line PD-1/PD-L1 inhibitors at Peking Union Medical College Hospital between May 2018 and April 2022. Immune cell counts were determined by flow cytometry.

**Results:**

Patients who responded to PD-1/PD-L1 inhibitors had significantly higher circulating CD8+CD28+ T-cell counts (median [range] count: 236 [30-536] versus 138 [36-460]/μL, p < 0.001). Using 190/μL as the cutoff value, the sensitivity and specificity of CD8+CD28+ T cells for predicting immunotherapy response were 0.689 and 0.714, respectively. Furthermore, the median progression-free survival (PFS, not reached versus 8.7 months, p < 0.001) and overall survival (OS, not reached versus 16.2 months, p < 0.001) were significantly longer in the patients with higher CD8+CD28+ T-cell counts. However, the CD8+CD28+ T-cell level was also associated with the incidence of grade 3-4 immune-related adverse events (irAEs). The sensitivity and specificity of CD8+CD28+ T cells for predicting irAEs of grade 3-4 were 0.846 and 0.667, respectively, at the threshold of CD8+CD28+ T cells ≥ 309/μL.

**Conclusions:**

High circulating CD8+CD28+ T-cell levels is a potential biomarker for immunotherapy response and better prognosis, while excessive CD8+CD28+ T cells (≥ 309/μL) may also indicate the emergence of severe irAEs.

## 1 Introduction

PD-1/PD-L1 (programmed cell death-1/programmed cell death-ligand 1) inhibitors, known as a kind of immune checkpoint inhibitors (ICIs), have revolutionized the paradigm of tumor therapy (1). PD-1/PD-L1 inhibitors work by abrogating the immune tolerance of T cells, resulting in the reactivation of immune cells and a subsequent antitumor response (1). However, the overall response rate to ICI treatment is only approximately 30% across malignancies (1, 2). Therefore, it is necessary to explore biomarkers to anticipate which patients will benefit from ICI therapy and reduce unwanted toxicities and costs.

Several studies have proposed PD-L1 expression in the tumor microenvironment (TME) and tumor-infiltrating lymphocytes (TILs) as cancer immunotherapy response biomarkers (2, 3). Nevertheless, it is generally difficult to obtain sufficient samples from tissue biopsy to delineate the heterogeneity of the tumor (4). On the other hand, tumor cells can reshape the immune environment at the tumor site and result in systemic effects (5). Immune cells derived from peripheral blood can eventually infiltrate the TME and may provide information in the use of ICI therapy (6). Some studies have found a good correlation between immune cell profiles in peripheral blood and tumor tissue (4, 7). Considering the accessibility of noninvasive liquid biopsies, it is advisable to employ peripheral blood lymphocyte subsets to predict immunotherapy outcomes.

In the present retrospective cohort study, we aimed to explore the correlation between circulating lymphocyte profiles and immunotherapy outcomes in cancer patients in the treatment of PD-1/PD-L1 inhibitors. To reduce confounding factors and mitigate the effect of front-line systematic treatment on immune cell profiles (8), we focused only on patients receiving first-line immunotherapy.

## 2 Materials and methods

### 2.1 Patients

We reviewed patients received PD-1/PD-L1 inhibitors in the Department of Medical Oncology, Peking Union Medical College Hospital (PUMCH) between May 2018 and April 2022 with the Electronic Medical Record Analytical Database (PUMCH-EMERALD). Inclusion criteria were as follows: 1) patients histopathologically diagnosed with cancers; 2) received at least 1 cycle of PD-1/PD-L1 inhibitors; and 3) available lymphocyte subset test within one month before the initiation of immunotherapy. The exclusion criteria were as follows: 1) received any systemic antitumor treatment before PD-1/PD-L1 inhibitor therapy; 2) died or lost of follow-up within one month before immunotherapy initiation; 3) survival outcomes or immune-related adverse events (irAEs) could not be assessed; and 4) any known second primary tumors. Immunotherapy outcomes were evaluated by medical records and telephone follow-up. Consent to participate was waived because of the deidentified data of the retrospective study.

### 2.2 Assessments

PD-1/PD-L1 inhibitors were administrated until tumor progression or unacceptable toxicity. Patients were followed up until loss of contact or death by October 21, 2022. Tumor assessment was carried out every 6 to 12 weeks using computed tomography (CT) scans or magnetic resonance imaging (MRI). Treatment responses were categorized as complete response (CR), partial response (PR), stable disease (SD), and progressive disease (PD) according to Response Evaluation Criteria in Solid Tumors version 1.1 (9). Patients who achieved CR or PR were considered immunotherapy responders, and the remainder were considered nonresponders. The objective response rate (ORR) was defined as the rate of best response of either CR or PR. Progression-free survival (PFS) was defined from the date of immunotherapy initiation to tumor progression or death due to any cause in the absence of progression. Overall survival (OS) was defined from the date of immunotherapy initiation to death due to any cause. The irAEs were graded based on the Common Terminology Criteria for Adverse Events version 5.0. The efficacy and irAEs were evaluated by two blinded independent senior clinical oncologists.

EDTA-anticoagulated peripheral whole blood was freshly collected before immunotherapy onset and tested with a panel of antibodies directed against antigen combinations of CD3/CD8/CD4, CD3/CD16CD56/CD19, CD28/CD8/CD4, HLA-DR/CD38/CD8, CD62L/CD45RA/CD4 and isotype controls (Immunotech, France). The circulating lymphocyte immunophenotype was determined by three-color flow cytometry (Epics XL flow cytometry; Bechman Coulter, USA) as previously described (10).

### 2.3 Statistical analysis

In this study, Mann−Whitney U test, Pearson’s chi-square test and Fisher’s exact test were utilized for continuous variables and categorical variables, respectively. Logistic regression was performed to explore variables associated with immunotherapy response and irAEs. The cutoff value of the CD8+CD28+ T-cell count for predicting immunotherapy response or irAEs was determined by the receiver operating characteristic (ROC) curve. Univariate and multivariate Cox analyses were conducted to identify variables associated with survival outcomes, and only the statistically significant factors in univariate analysis were selected during the multivariate analysis. Survival outcome was further estimated by the Kaplan−Meier method and log-rank test. Moreover, propensity-score matching (PSM) was used to reduce the influence of confounding factors. The propensity scores were calculated by cancer type, age and TNM stage. All statistical analyses were conducted using R software (version 3.6.1, https://www.r-project.org/). All p values were two-tailed; p < 0.05 was considered statistically significant.

## 3 Result

### 3.1 Patient characteristics

The main cancer types of the 87 enrolled patients were non-small cell lung cancer (NSCLC) and digestive tract cancers. The median age of the patients was 61 (range 32-85) years. The median follow-up time was 15.6 (range 2.5-49.4) months. None of the patients had a previous diagnosis of autoimmune disease. Eighty-three patients (95.4%) had an ECOG performance status score of 0 or 1, and 65 (74.7%) had stage IV disease (**Table 1**). The ORR of the population was 51.7%. The median PFS was 12.5 months, while the median OS was not reached. Moreover, 41 (47.1%) patients developed any grade irAEs, 9 (10.3%) patients experienced grade 3-4 irAEs, and no patients died due to irAEs.

**Table 1:** Baseline characteristics.

Variables	Total (n=87)
Age, median (range), years	61 (32-85)
Sex, male	63 (72.4%)
Tumor type	
Non-small cell lung cancer	26 (29.9%)
Gastric cancer	17 (19.5%)
Head and neck cancer	13 (14.9%)
Esophageal cell squamous carcinoma	12 (13.8%)
Others^a^	19 (21.8%)
Performance status	
0-1	83 (95.4%)
2-3	4 (4.6%)
TNM stage	
III	22 (25.3%)
IV	65 (74.7%)
Liver metastasis	20 (23.0%)
Multiple metastases	23 (26.4%)
PD-1/PD-L1 inhibitor	
Pembrolizumab	35 (40.2%)
Nivolumab	20 (23.0%)
Tislelizumab	11 (12.6%)
Toripalimab	10 (11.5%)
Others^b^	11 (12.6%)
Combination therapy^c^	79 (90.8%)
PD-L1 status	
Positive^d^	30 (34.5%)
Negative	6 (6.9%)
Unknown	51 (58.6%)
MSI status	
MSI-H	7 (8.0%)
MSS	31 (35.6%)
Unknown	49 (56.3%)
Lymphocytes, median (range),/μL	1500 (330-4170)
CD19+ B cells, median (range),/μL	106 (7-535)
CD16+CD56+ NK cells, median (range),/μL	234 (44-1360)
CD3+ T cells, median (range),/μL	1040 (228-2360)
CD3+CD4+ T cells, median (range),/μL	594 (113-1180)
CD3+CD8+ T cells, median (range),/μL	347 (96-1370)
CD4+CD45RA- T cells, median (range),/μL	462 (80-987)
CD4+CD45RA+ T cells, median (range),/μL	125 (14-578)
CD4+CD45RA+CD62L+ T cells, median (range),/μL	115 (13-564)
CD4+CD28+ T cells, median (range),/μL	556 (109-1180)
CD8+CD28+ T cells, median (range),/μL	187 (30-536)
CD8+HLA-DR+ T cells, median (range),/μL	167 (38-949)
CD8+CD38+ T cells, median (range),/μL	132 (43-736)
CD4+/CD8+	1.62 (0.2-6.63)

MSI, microsatellite instability; MSI-H, MSI-high; MSS, microsatellite-stable; PD-L1, programmed death ligand-1.

a Five patients had urological cancer, 4 had colorectal cancer, 3 had small cell lung cancer, 2 had periampullary carcinoma, 1 had hepatocellular carcinoma, 1 had cholangiocarcinoma, 1 had endometrial cancer, 1 had cervical cancer, and 1 had cutaneous squamous cell carcinoma.

b Four patients were treated with durvalumab, 3 with camrelizumab, 2 with sintilimab, 1 with atezolizumab, and 1 with penpulimab.

c 71 patients treated with combined chemotherapy, 6 with combined targeted therapy, 1 with combined chemotherapy plus targeted therapy, and 1 with combined ipilimumab.

d PD-L1 combined positive score ≥ 1 or tumor proportion score ≥ 1%.

### 3.2 Evaluation of efficacy

**Figure 1:**
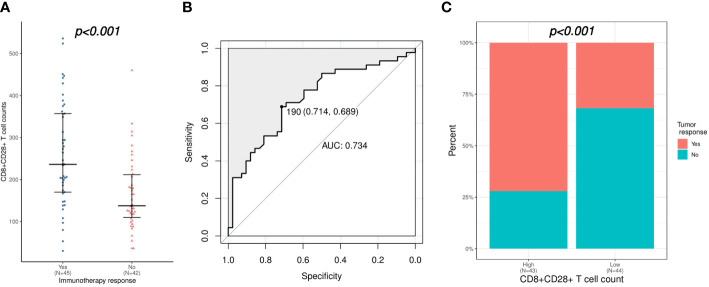
Correlation of CD8+CD28+ T-cell count with the immunotherapy response. **(A)** Comparison of CD8+CD28+ T-cell counts between immunotherapy responders and nonresponders. **(B)** Validation of the predictive value of the CD8+CD28+ T-cell count for immunotherapy response using an ROC curve. **(C)** Comparison of immunotherapy response between patients with high (≥ 190 cells/μL) and low (< 190 cells/μL) CD8+CD28+ T-cell counts. ROC, receiver operating characteristic.

Patients were categorized as immunotherapy responders (CR or PR, n = 45) or nonresponders (SD or PD, n = 42), in order to investigate the association between baseline peripheral lymphocyte subsets level and immunotherapy response. As shown in **Table 2**, univariate logistic regression analysis demonstrated that total lymphocytes, CD16+CD56+ NK cells, CD8+CD28+ T cells, and CD8+CD38+ T cells were identified as potential indicators of immunotherapy response. Further multivariate analysis confirmed that a higher CD8+CD28+ T-cell count (odds ratio [OR]: 1.009, 95% confidence interval [CI]: 1.002-1.016, p = 0.006) was an independent predictor of immunotherapy response. Consistently, CD8+CD28+ T-cell counts were significantly higher in immunotherapy responders than in nonresponders (median [range] count: 236 [30-536] versus 138 [36-460]/μL, p < 0.001; **Figure 1A**). Furthermore, the CD8+CD28+ T-cell count was a desirable predictor of immunotherapy response (area under the curve [AUC]: 0.734), with a specificity of 0.714 and sensitivity of 0.689 using CD8+CD28+ T cells ≥ 190/μL as the threshold (**Figure 1B**). On the other hand, patients with high CD8+CD28+ T-cell counts (≥190/μL) had a significantly higher ORR (72.1% versus 31.8%, p < 0.001; **Figure 1C**).

**Table 2:** Univariate and multivariate logistic regression for immunotherapy response, irAEs of any grade, and irAEs of grade 3-4.

Variables	Treatment response (P value, OR)		irAEs (P value, OR)		Severe irAEs (P value, OR)	
	Univariate	Multivariate	Univariate	Multivariate	Univariate	Multivariate
Age (years)	0.676, 1.008	–	0.762, 1.006	–	0.459, 1.025	–
Sex (Male vs. Female)	0.249, 1.750	–	0.418, 0.677	–	0.705, 1.375	–
Performance status (2-3 vs. 0-1)	0.361, 2.929	–	0.383, 0.358	–	0.994, 0.000	–
Tumor type (ESCC vs. NSCLC)	0.970, 1.027	–	0.487, 0.612	–	0.766, 0.697	–
Tumor type (GC vs. NSCLC)	0.292, 0.513	–	0.418, 0.600	–	0.574, 1.643	–
Tumor type (Head and neck vs. NSCLC)	0.497, 0.629	–	0.368, 0.536	–	0.711, 0.639	–
Tumor type (Others vs. NSCLC)	0.736, 0.815	–	0.936, 0.952	–	0.476, 0.423	–
TNM stage (IV vs. III)	0.425, 0.671	–	0.755, 0.857	–	0.175, 0.375	–
Liver metastasis (Yes vs. No)	0.738, 1.186	–	0.086, 0.392	–	0.387, 0.388	–
Multiple metastases (Yes vs. No)	0.358, 0.637	–	0.683, 0.819	–	0.293, 0.318	–
Immunotherapy (Nivolumab vs. Pembrolizumab)	0.508, 0.689	–	0.415, 0.630	–	0.123, 4.125	–
Immunotherapy (Tislelizumab vs. Pembrolizumab)	0.586, 1.474	–	0.857, 1.133	–	0.224, 3.667	–
Immunotherapy (Toripalimab vs. Pembrolizumab)	0.185, 0.361	–	0.526, 0.630	–	0.994, 0.000	–
Immunotherapy (Others vs. Pembrolizumab)	0.586, 1.474	–	0.730, 0.787	–	0.695, 1.650	–
Combination therapy (Yes vs. No)	0.525, 0.615	–	0.117, 0.265	–	0.994, 1479	–
PD-L1 status (Positive vs. Negative)	0.190, 3.454	–	0.244, 3.000	–	0.992, 8537	–
MSI status (MSS vs. MSI-H)	0.350, 0.427	–	0.676, 0.703	–	0.721, 0.643	–
Lymphocytes (/μL)	**0.039**, 1.001	0.161, 0.999	0.149, 0.999	–	0.845, 1.000	–
CD19+ B cells (/μL)	0.447, 1.002	–	0.616, 0.999	–	0.376, 0.996	–
CD16+CD56+ NK cells (/μL)	**0.022**, 1.003	0.072, 1.003	0.890, 1.000	–	0.160, 1.002	–
CD3+ T cells (/μL)	0.143, 1.001	–	0.061, 0.999	–	0.736, 1.000	–
CD3+CD4+ T cells (/μL)	0.809, 1.000	–	**0.034**, 0.998	0.714, 1.002	0.498, 0.999	–
CD3+CD8+ T cells (/μL)	0.060, 1.002	–	0.133, 0.999	–	0.857, 1.000	–
CD4+CD45RA- T cells (/μL)	0.265, 1.001	–	0.063, 0.998	–	0.336, 0.998	–
CD4+CD45RA+ T cells (/μL)	0.138, 0.997	–	0.170, 0.997	–	0.801, 1.001	–
CD4+CD45RA+CD62L+ T cells (/μL)	0.086, 0.996	–	0.199, 0.997	–	0.683, 1.001	–
CD4+CD28+ T cells (/μL)	0.964, 1.000	–	**0.025**, 0.998	0.377, 0.996	0.510, 0.999	–
CD8+CD28+ T cells (/μL)	**0.001**, 1.008	**0.006**, 1.009	0.799, 1.000	–	**0.038**, 1.006	**0.038**, 1.006
CD8+HLA-DR+ T cells (/μL)	0.125, 1.002	–	0.551, 0.999	–	0.727, 0.999	–
CD8+CD38+ T cells (/μL)	**0.042**, 1.006	0.162, 1.004	0.845, 1.000	–	0.671, 1.001	–
CD4+/CD8+	0.058, 0.631	–	0.376, 0.828	–	0.282, 0.600	–

ESCC, esophageal cell squamous carcinoma; GC, gastric cancer; irAEs, immune-related adverse events; MSI, microsatellite instability; MSI-H, MSI-high; MSS, microsatellite-stable; NSCLC, non-small cell lung cancer; PD-L1, programmed death ligand-1. The bold values indicate they are statistically significant (p<0.05).

**Figure 2:**
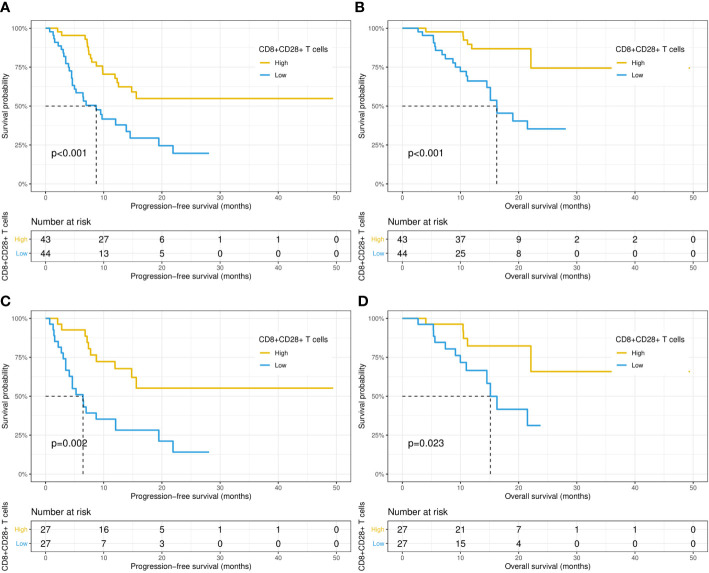
Kaplan−Meier curves of progression-free survival and overall survival in patients with high (≥ 190 cells/μL) and low (< 190 cells/μL) CD8+CD28+ T-cell counts before **(A, B)** and after **(C, D)** propensity score matching.

Then, the relationship between the survival outcomes and circulating lymphocyte subsets of the patients was analyzed. The univariate Cox regression analysis suggested that liver metastasis and CD8+CD28+ T-cell count were indicators of PFS in patients taking PD-1/PD-L1 inhibitors (**Table 3**). Multivariate analysis showed that a higher CD8+CD28+ T-cell count (HR: 1.00, 95% CI: 0.99-1.00, p = 0.002) was an independent predictor of better PFS. Likewise, multivariate Cox analysis identified that the CD8+CD28+ T-cell count (HR: 0.99, 95% CI: 0.99-1.00, p = 0.018) was also significantly correlated with OS (**Table 4**). Furthermore, it suggested that the median PFS (not reached versus 8.7 months, p < 0.001) and median OS (not reached versus 16.2 months, p < 0.001) were significantly longer in the higher CD8+CD28+ T-cell group (≥ 190/μL) in Kaplan-Meier curves (**Figures 2A, B**). By using PSM, the CD8+CD28+ T-cell count was robustly related to with the median PFS and median OS of the patients (**Figures 2C, D**).

**Table 3:** Univariate and multivariate Cox analyses of factors for progression-free survival.

Variables	Univariate analysis		Multivariate analysis	
	HR (95% CI)	P value	HR (95% CI)	P value
Age (years)	0.98 (0.95,1)	0.081	-	-
Sex (Male vs. Female)	0.71 (0.38,1.33)	0.286	-	-
Tumor (ESCC vs. NSCLC)	1.24 (0.49,3.14)	0.657	-	-
Tumor (GC vs. NSCLC)	1.83 (0.76,4.38)	0.174	-	-
Tumor (Head and neck vs. NSCLC)	1.3 (0.53,3.19)	0.566	-	-
Tumor (Others vs. NSCLC)	1.25 (0.54,2.91)	0.603	-	-
Performance status (2-3 vs. 0-1)	0.60 (0.08,4.36)	0.613	-	-
TNM stage (IV vs. III)	1.69 (0.81,3.53)	0.159	-	-
Liver metastasis (Yes vs. No)	2.08 (1.09,3.99)	**0.027**	1.48(0.75,2.93)	0.263
Multiple metastases (Yes vs. No)	1.74 (0.93,3.26)	0.082	-	-
Immunotherapy (Nivolumab vs. Pembrolizumab)	1.34 (0.62,2.90)	0.459	-	-
Immunotherapy (Tislelizumab vs. Pembrolizumab)	0.58 (0.20,1.70)	0.319	-	-
Immunotherapy (Toripalimab vs. Pembrolizumab)	1.61 (0.67,3.82)	0.285	-	-
Immunotherapy (Others vs. Pembrolizumab)	1.15 (0.46,2.88)	0.765	-	-
Combination therapy (Yes vs. No)	1.26 (0.45,3.55)	0.661	-	-
PD-L1 status (Positive vs. Negative)	0.54 (0.18,1.62)	0.269	-	-
MSI status (MSS vs. MSI-H)	2.53 (0.59,10.90)	0.214	-	-
Lymphocytes (/μL)	1 (1,1)	0.086	-	-
CD19+ B cells (/μL)	1 (1,1)	0.570	-	-
CD16+CD56+ NK cells (/μL)	1 (1,1)	0.219	-	-
CD3+ T cells (/μL)	1 (1,1)	0.120	-	-
CD3+CD4+ T cells (/μL)	1 (1,1)	0.252	-	-
CD3+CD8+ T cells (/μL)	1 (1,1)	0.232	-	-
CD4+CD45RA- T cells (/μL)	1 (1,1)	0.122	-	-
CD4+CD45RA+ T cells (/μL)	1 (1,1)	0.803	-	-
CD4+CD45RA+CD62L+ T cells (/μL)	1 (1,1)	0.579	-	-
CD4+CD28+ T cells (/μL)	1 (1,1)	0.352	-	-
CD8+CD28+ T cells (/μL)	0.99 (0.99,1)	**0.001**	1(0.99,1)	**0.002**
CD8+HLA-DR+ T cells (/μL)	1 (1,1)	0.173	-	-
CD8+CD38+ T cells (/μL)	1 (1,1)	0.272	-	-
CD4+/CD8+	1.11 (0.87,1.41)	0.394	-	-

ESCC, esophageal cell squamous carcinoma; GC, gastric cancer; MSI, microsatellite instability; MSI-H, MSI-high; MSS, microsatellite-stable; NSCLC, non-small cell lung cancer; PD-L1, programmed death ligand-1. The bold values indicate they are statistically significant (p< 0.05).

**Table 4:** Univariate and multivariate Cox analyses of factors for overall survival.

Variables	Univariate analysis		Multivariate analysis	
	HR (95% CI)	P value	HR (95% CI)	P value
Age (years)	1 (0.97,1.03)	0.946	-	-
Sex (Male vs. Female)	0.96 (0.42,2.22)	0.928	-	-
Tumor (ESCC vs. NSCLC)	1.97 (0.52,7.38)	0.317	4.83 (0.95,24.6)	0.058
Tumor (GC vs. NSCLC)	4.01 (1.25,12.89)	**0.020**	3.06 (0.58,16.14)	0.187
Tumor (Head and neck vs. NSCLC)	1.26 (0.34,4.70)	0.735	0.40 (0.05,2.98)	0.369
Tumor (Others vs. NSCLC)	1.53 (0.46,5.07)	0.483	0.89 (0.25,3.25)	0.864
Performance status (2-3 vs. 0-1)	2.03 (0.27,15.33)	0.493	-	-
TNM stage (IV vs. III)	4 (0.94,16.93)	0.060	-	-
Liver metastasis (Yes vs. No)	1.89 (0.79,4.54)	0.154	-	-
Multiple metastases (Yes vs. No)	1.61 (0.71,3.62)	0.251	-	-
Immunotherapy (Nivolumab vs. Pembrolizumab)	2.71 (1,7.32)	**0.049**	1.34 (0.29,6.12)	0.703
Immunotherapy (Tislelizumab vs. Pembrolizumab)	0.34 (0.04,2.76)	0.315	0.24 (0.02,2.44)	0.228
Immunotherapy (Toripalimab vs. Pembrolizumab)	1.79 (0.58,5.47)	0.309	5.65 (0.94,33.92)	0.058
Immunotherapy (Others vs. Pembrolizumab)	1.87 (0.56,6.28)	0.308	2.96 (0.79,11.10)	0.109
Combination therapy (Yes vs. No)	1.56 (0.36,6.70)	0.552	-	-
PD-L1 status (Positive vs. Negative)	0.27 (0.08,0.94)	**0.039**	0.23 (0.05,0.99)	**0.049**
MSI status (MSS vs. MSI-H)	94048932 (0,Inf)	0.997	-	-
Lymphocytes (/μL)	1 (1,1)	0.078	-	-
CD19+ B cells (/μL)	1 (0.99,1)	0.426	-	-
CD16+CD56+ NK cells (/μL)	1 (1,1)	0.078	-	-
CD3+ T cells (/μL)	1 (1,1)	0.190	-	-
CD3+CD4+ T cells (/μL)	1 (1,1)	0.117	-	-
CD3+CD8+ T cells (/μL)	1 (1,1)	0.731	-	-
CD4+CD45RA- T cells (/μL)	1 (1,1)	0.093	-	-
CD4+CD45RA+ T cells (/μL)	1 (0.99,1)	0.637	-	-
CD4+CD45RA+CD62L+ T cells (/μL)	1 (1,1)	0.873	-	-
CD4+CD28+ T cells (/μL)	1 (1,1)	0.212	-	-
CD8+CD28+ T cells (/μL)	0.99 (0.99,1)	**0.003**	0.99 (0.99,1)	**0.018**
CD8+HLA-DR+ T cells (/μL)	1 (1,1)	0.926	-	-
CD8+CD38+ T cells (/μL)	1 (0.99,1)	0.387	-	-
CD4+/CD8+	1.05 (0.75,1.47)	0.763	-	

ESCC, esophageal cell squamous carcinoma; GC, gastric cancer; MSI, microsatellite instability; MSI-H, MSI-high; MSS, microsatellite-stable; NSCLC, non-small cell lung cancer; PD-L1, programmed death ligand-1.

The bold values indicate they are statistically significant (p<0.05).

### 3.3 Safety analysis

**Figure 3:**
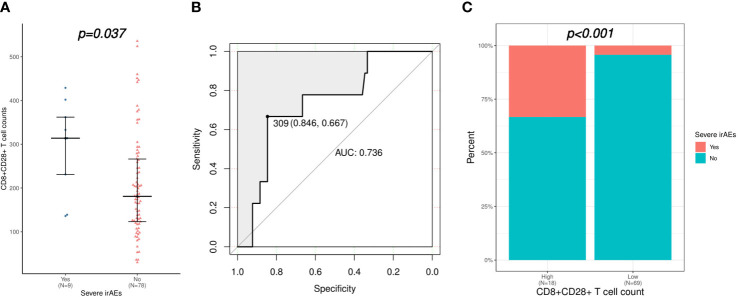
Correlation of CD8+CD28+ T-cell count with severe irAEs (irAEs of grade 3-4). **(A)** Comparison of CD8+CD28+ T-cell counts between patients with or without severe irAEs. **(B)** Validation of the predictive value of the CD8+CD28+ T-cell count for severe irAEs using an ROC curve. **(C)** Comparison of severe irAEs between patients with high (≥ 309 cells/μL) and low (< 309 cells/μL) CD8+CD28+ T-cell counts. irAEs, immune-related adverse events; ROC, receiver operating characteristic.

As shown in **Table 2**, the univariate logistic regression analysis suggested that CD3+CD4+ T cells and CD4+CD28+ T cells were associated with the occurrence of irAEs of any grade, while further multivariate analysis showed no credible predictor for irAEs. However, the logistic regression analysis demonstrated that only higher CD8+CD28+ T-cell level was a risk factor for severe irAEs (OR: 1.006, 95% CI: 1.000-1.011, p = 0.038). Accordingly, CD8+CD28+ T-cell counts were significantly higher in patients who experienced severe irAEs (median [range] count: 314 [136-429] versus 181 [30-536]/μL, p = 0.037; **Figure 3A**). The AUC was 0.736; at the threshold of CD8+CD28+ T cells ≥ 309/μL, the specificity was 0.846, and the sensitivity was 0.667 (**Figure 3B**). Moreover, severe irAEs occurred more often in patients with excessive CD8+CD28+ T-cell counts (≥ 309/μL) (33.3% versus 4.3%, p < 0.001; **Figure 3C**).

### 3.4 Patient classification based on CD8+CD28+ T-cell level

**Figure 4:**
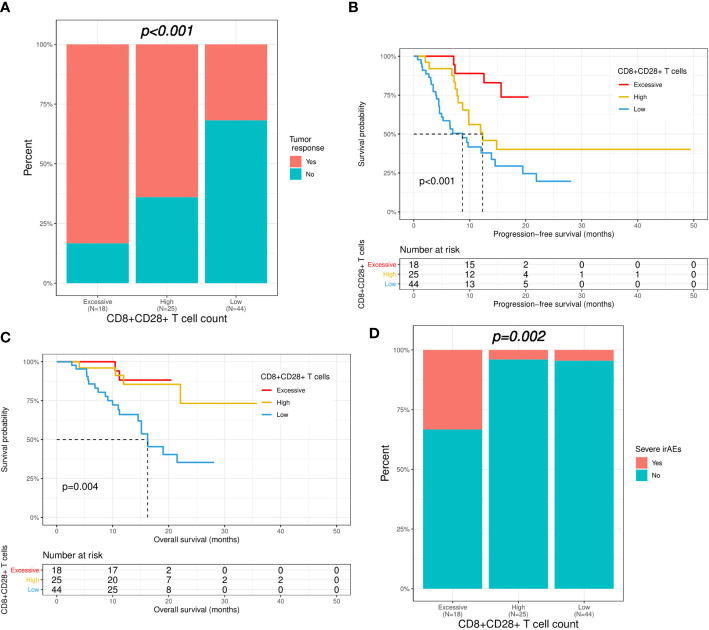
Correlation of CD8+CD28+ T-cell count with immunotherapy efficacy and safety. Patients were divided into three groups based on the level of CD8+CD28+ T cells (excessive: ≥ 309; high: 309-190; low: <190). **(A)** Comparison of immunotherapy response between the three groups. **(B, C)** Kaplan−Meier curves of progression-free survival and overall survival in the three groups. **(D)** Comparison of severe irAEs between the three groups. irAEs, immune-related adverse events.

According to the results of the analysis above, all patients were divided into three groups based on the level of CD8+CD28+ T cells (excessive: ≥ 309/μL; high: 309-190/μL; low: <190/μL). Compared with patients with a low CD8+CD28+ T-cell level, patients with an excessive or high level of CD8+CD28+ T cells had a significantly higher ORR (83.3% versus 64.0% versus 31.8%, p < 0.001; **Figure 4A**). Furthermore, the median PFS (not reached versus 12.3 versus 8.7 months, p < 0.001; **Figure 4B**) and OS (not reached versus not reached versus 16.2 months, p = 0.004; **Figure 4C**) were also significantly longer in the patients with an excessive or high level of CD8+CD28+ T cells. Nevertheless, severe irAEs also occurred more often in patients with an excessive level of CD8+CD28+ T cells (≥ 309/μL) than in those with a high or low level of CD8+CD28+ T cells (33.3% versus 4.2% versus 4.5%, p = 0.002; **Figure 4D**).

## 4 Discussion

Although clinicians are not very satisfied with the accessibility and accuracy of PD-L1 expression and tumor-infiltrating immune cells in predicting the efficacy of PD-1/PD-L1 inhibitors, there is still a lack of convenient and reliable peripheral blood-derived markers to predict the efficacy and safety of immunotherapy. In the current study, we evaluated the relationship between circulating lymphocytes and immunotherapy outcomes in cancer patients receiving first-line PD-1/PD-L1 inhibitors.

Although it was found that patients who responded to immunotherapy tended to have reduced baseline circulating T cells in comparison with nonresponders (11). As the main effector in tumor immunity (12), many previous studies have confirmed that a higher level of tumor-infiltrating CD8+ T cells was associated with the immunotherapy response (13–15). Consistently, accumulating evidence suggests that the level of circulating CD8+ T cells is also a more accessible biomarker to predict immunotherapy efficacy (16, 17), which suggests that more attention should be given to circulating CD8+ T cells when exploring the correlation between immune cells and immunotherapy outcomes.

CD28 is a pivotal costimulatory molecule that activates effector T cells and induces antitumor immunity by competing with cytotoxic T-lymphocyte associated protein 4 (CTLA-4) for B7-1 and B7-2 ligands (18). It has been proven that CD28/B7 pathway blockade or CD28 deficiency eliminates effector T-cell expansion and the antitumor effect of PD-1 inhibitors (19, 20). Expression of CD28/PD-1 fusion proteins on CD8+ T cells can also overcome the immunosuppressive effect induced by the PD-1/PD-L1 axis and enhance cytolytic activity (21). Furthermore, patients with ovarian cancer had a lower level of circulating CD8+CD28+ T cells (22). Increased CD8+CD28+ T cells indicates a better early response to radiotherapy and favorable survival outcomes in NSCLC patients (23, 24). Our results suggest that circulating CD8+CD28+ T-cell level was associated with immunotherapy efficacy and survival outcomes, even though the impact of confounding factors was minimized. Considering the role of CD28 and the association between enhanced T-cell activation and irAEs (25), it is understandable that a higher CD8+CD28+ T-cell level can also predict the development of severe irAEs. Indeed, a recent study supported that upregulated CD28 was correlated with grade 3-5 irAEs (26). Selective CD28 antagonists are currently being tried in the treatment of autoimmune diseases, confirming their role in suppressing overactivated autoimmunity (27). As a desirable indicator of immunotherapy efficacy and safety, we further categorized patients into three groups according to circulating CD8+CD28+ T cells and confirmed that patients with CD8+CD28+ T-cell counts between 190 and 309/μL had a decent immunotherapy response but fewer severe irAEs. This could help anticipate patients who are befitting candidates for immunotherapy.

Noninvasive repeated sampling of peripheral blood makes dynamic monitoring of circulating lymphocyte profiles a remarkable field. Wang et al. (28) demonstrated that the increase in CD8+ eomesodermin (EOMES)+ and CD8+ EOMES+granzyme B+ T cells, as well as the decline in CD4+ EOMES+Ki67+ T cells after ipilimumab, were associated with melanoma relapse. The decrease in CD8+Ki67+ T cells can also indicate the emergence of irAEs. Tada et al. (29) reported that increased CD4+ and CD8+ terminal effector memory T cells were associated with the response to nivolumab in patients with head and neck squamous cell carcinoma, while another study suggested that the decrease in CD4+ T cells after the first dose of ICIs was a poor predictor for OS and tumor progression (30). Regrettably, there were only 16 patients who had dynamic data on the circulating lymphocyte profile in our cohort; therefore, it was unable to examine the influence of dynamic circulating lymphocytes on the safety and efficacy of ICIs. We will pay more attention to the significance of the dynamic changes in circulating lymphocyte profiles in patients treated with immunotherapy in the future.

This is the first study to evaluate the effect of circulating CD8+CD28+ T cells on ICI administration in the first-line setting to the best of our knowledge. The results add to the growing evidence supporting the role of circulating lymphocytes in patients receiving immunotherapy. However, there are several limitations in our research. First, there may be potential selection bias in the retrospective study. Second, the relatively small size and single-center approach may confine the generalization of our results to other situations. Third, given the complexity of cell lineages, solely relying on the limited cell surface markers used in this study may not be sufficient to elucidate the role of immune cells in antitumor immunity. In the future, more prospective trials and preclinical studies using more detailed surface markers are expected to clarify the role of circulating immune cells in immunotherapy. Moreover, as mentioned above, the dynamic change in lymphocytes and the abundance of lymphocytes in tumor tissues may reflect the change in patients’ antitumor immunity, but we failed to analyze the effect of dynamic circulating lymphocytes on the safety and efficacy of PD-1/PD-L1 inhibitors.

## 5 Conclusion

In summary, our data suggested that a high circulating CD8+CD28+ T-cell level indicates an immunotherapy response and prolonged survival, but excessive CD8+CD28+ T cells (≥ 309/μL) may also indicate the risk of severe irAEs.

## Data availability statement

The raw data supporting the conclusions of this article will be made available by the authors, without undue reservation.

## Ethics statement

This study was approved by the Medical Ethics Committee of Peking Union Medical College Hospital (S-K2098) and carried out following the Helsinki Declaration on experimentation involving human subjects. Written informed consent for participation was not required for this study in accordance with the national legislation and the institutional requirements.

## Author contributions

RG, HT, and YW conceived the research. RG and HT carried out the literature search. RG, HT, TY, SL, ZL, YL, WQ, NZ, NL, YG, FG, and YS conducted data collection and analysis. RG and HT participated in data visualization. RG and HT drafted the manuscript, and TL, CB, and YW reviewed the manuscript. All authors contributed to the article and approved the submitted version.

## References

[1] Bagchi S Yuan R Engleman EG. Immune checkpoint inhibitors for the treatment of cancer: Clinical impact and mechanisms of response and resistance. Annu Rev Pathol (2021) 16:223–49. doi: 10.1146/annurev-pathol-042020-042741 33197221

[2] Doroshow DB Bhalla S Beasley MB Sholl LM Kerr KM Gnjatic S. PD-L1 as a biomarker of response to immune-checkpoint inhibitors. Nat Rev Clin Oncol (2021) 18(6):345–62. doi: 10.1038/s41571-021-00473-5 33580222

[3] Gonzalez-Ericsson PI Stovgaard ES Sua LF Reisenbichler E Kos Z Carter JM. The path to a better biomarker: Application of a risk management framework for the implementation of PD-L1 and TILs as immuno-oncology biomarkers in breast cancer clinical trials and daily practice. J Pathol (2020) 250(5):667–84. doi: 10.1002/path.5406 32129476

[4] . Intratumoral versus circulating lymphoid cells as predictive biomarkers in lung cancer patients treated with immune checkpoint inhibitors: Is the easiest path the best one? Cells. (2020) 9(6):1525. doi: 10.3390/cells9061525 32580514 PMC7348938

[5] Liang H Chu X Zhao J Xing G Si Y. Elevated peripheral blood b lymphocytes and CD3(+)CD4(-)CD8(-) T lymphocytes in patients with non-small cell lung cancer: A preliminary study on peripheral immune profile. Oncol Lett (2018) 15(6):8387–95. doi: 10.3892/ol.2018.8424 PMC5950528 29805573

[6] Araujo B Hansen M Spanggaard I Rohrberg K Reker Hadrup S Lassen U. Immune cell profiling of peripheral blood as signature for response during checkpoint inhibition across cancer types. Front Oncol (2021) 11:558248. doi: 10.3389/fonc.2021.558248 33842304 PMC8027233

[7] Kim KH Kim CG Shin EC. Peripheral blood immune cell-based biomarkers in anti-PD-1/PD-L1 therapy. Immune Netw (2020) 20(1):e8. doi: 10.4110/in.2020.20.e8 32158596 PMC7049582

[8] Niu M Combs SE Linge A Krause M Baumann M Lohaus F. Comparison of the composition of lymphocyte subpopulations in non-relapse and relapse patients with squamous cell carcinoma of the head and neck before, during radiochemotherapy and in the follow-up period: A multicenter prospective study of the German cancer consortium radiation oncology group (DKTK-ROG). Radiat Oncol (2021) 16(1):141. doi: 10.1186/s13014-021-01868-5 34332614 PMC8325802

[9] Eisenhauer EA Therasse P Bogaerts J Schwartz LH Sargent D Ford R. New response evaluation criteria in solid tumours: Revised RECIST guideline (version 1.1). Eur J Cancer (2009) 45(2):228–47. doi: 10.1016/j.ejca.2008.10.026 19097774

[10] Qin L Jing X Qiu Z Cao W Jiao Y Routy JP. Aging of immune system: Immune signature from peripheral blood lymphocyte subsets in 1068 healthy adults. Aging (Albany NY) (2016) 8(5):848–59. doi: 10.18632/aging.100894 PMC4931839 26886066

[11] Krieg C Nowicka M Guglietta S Schindler S Hartmann FJ Weber LM. High-dimensional single-cell analysis predicts response to anti-PD-1 immunotherapy. Nat Med (2018) 24(2):144–53. doi: 10.1038/nm.4466 29309059

[12] Iwahori K. Cytotoxic CD8(+) lymphocytes in the tumor microenvironment. Adv Exp Med Biol (2020) 1224:53–62. doi: 10.1007/978-3-030-35723-8_4 32036604

[13] Peña-Asensio J Calvo H Torralba M Miquel J Sanz-de-Villalobos E Larrubia JR. Anti-PD-1/PD-L1 based combination immunotherapy to boost antigen-specific CD8(+) T cell response in hepatocellular carcinoma. Cancers (Basel) (2021) 13(8):1922. doi: 10.3390/cancers13081922 33923463 PMC8073815

[14] Siddiqui I Schaeuble K Chennupati V Fuertes Marraco SA Calderon-Copete S Pais Ferreira D. Intratumoral Tcf1(+)PD-1(+)CD8(+) T cells with stem-like properties promote tumor control in response to vaccination and checkpoint blockade immunotherapy. Immunity (2019) 50(1):195–211.e110. doi: 10.1016/j.immuni.2018.12.021 30635237

[15] Yang Z Deng Y Cheng J Wei S Luo H Liu L. Tumor-infiltrating PD-1(hi)CD8(+)-T-Cell signature as an effective biomarker for immune checkpoint inhibitor therapy response across multiple cancers. Front Oncol (2021) 11:695006. doi: 10.3389/fonc.2021.695006 34604032 PMC8479164

[16] Simon S Voillet V Vignard V Wu Z Dabrowski C Jouand N. PD-1 and TIGIT coexpression identifies a circulating CD8 T cell subset predictive of response to anti-PD-1 therapy. J Immunother Cancer. (2020) 8(2):e001631. doi: 10.1136/jitc-2020-001631 33188038 PMC7668369

[17] Kamphorst AO Pillai RN Yang S Nasti TH Akondy RS Wieland A. Proliferation of PD-1+ CD8 T cells in peripheral blood after PD-1-targeted therapy in lung cancer patients. Proc Natl Acad Sci USA (2017) 114(19):4993–8. doi: 10.1073/pnas.1705327114 PMC5441721 28446615

[18] Shirinbak S Chan RY Shahani S Muthugounder S Kennedy R Hung LT. Combined immune checkpoint blockade increases CD8+CD28+PD-1+ effector T cells and provides a therapeutic strategy for patients with neuroblastoma. Oncoimmunology (2021) 10(1):1838140. doi: 10.1080/2162402x.2020.1838140 33489468 PMC7801125

[19] Kamphorst AO Wieland A Nasti T Yang S Zhang R Barber DL. Rescue of exhausted CD8 T cells by PD-1-targeted therapies is CD28-dependent. Science (2017) 355(6332):1423–7. doi: 10.1126/science.aaf0683 PMC5595217 28280249

[20] Hui E Cheung J Zhu J Su X Taylor MJ Wallweber HA. T Cell costimulatory receptor CD28 is a primary target for PD-1-mediated inhibition. Science. (2017) 355(6332):1428–33. doi: 10.1126/science.aaf1292 PMC6286077 28280247

[21] Kobold S Grassmann S Chaloupka M Lampert C Wenk S Kraus F. Impact of a new fusion receptor on PD-1-Mediated immunosuppression in adoptive T cell therapy. J Natl Cancer Inst (2015) 107(8):djv146. doi: 10.1093/jnci/djv146 26105028 PMC4609553

[22] Ye S Chen W Zheng Y Wu Y Xiang L Li T. Peripheral lymphocyte populations in ovarian cancer patients and correlations with clinicopathological features. J Ovarian Res (2022) 15(1):43. doi: 10.1186/s13048-022-00977-3 35410290 PMC8996636

[23] Liu C Hu Q Hu K Su H Shi F Kong L. Increased CD8+CD28+ T cells independently predict better early response to stereotactic ablative radiotherapy in patients with lung metastases from non-small cell lung cancer. J Transl Med (2019) 17(1):120. doi: 10.1186/s12967-019-1872-9 30971280 PMC6458628

[24] Liu C Jing W An N Li A Yan W Zhu H. Prognostic significance of peripheral CD8+CD28+ and CD8+CD28- T cells in advanced non-small cell lung cancer patients treated with chemo(radio)therapy. J Transl Med (2019) 17(1):344. doi: 10.1186/s12967-019-2097-7 31623615 PMC6796409

[25] Kennedy LB Salama AKS. A review of cancer immunotherapy toxicity. CA Cancer J Clin (2020) 70(2):86–104. doi: 10.3322/caac.21596 31944278

[26] Wang Y Zou J Li Y Jiao X Wang Y Zhuo N. Serological biomarkers predict immune-related adverse events and clinical benefit in patients with advanced gastrointestinal cancers. Front Immunol (2022) 13:987568. doi: 10.3389/fimmu.2022.987568 36159840 PMC9492966

[27] Vanhove B Poirier N Fakhouri F Laurent L t Hart B Papotto PH. Antagonist anti-CD28 therapeutics for the treatment of autoimmune disorders. Antibodies (Basel) (2017) 6(4):19. doi: 10.3390/antib6040019 31548534 PMC6698823

[28] Wang W Yu D Sarnaik AA Yu B Hall M Morelli D. Biomarkers on melanoma patient T cells associated with ipilimumab treatment. J Transl Med (2012) 10:146. doi: 10.1186/1479-5876-10-146 22788688 PMC3527361

[29] Tada H Takahashi H Yamada K Masuda K Nagata Y Uchida M. Dynamic alterations of circulating T lymphocytes and the clinical response in patients with head and neck squamous cell carcinoma treated with nivolumab. Cancer Immunol Immunother (2022) 71(4):851–63. doi: 10.1007/s00262-021-03042-y PMC10992903 34463793

[30] Liu C Wang Y Li S Jiao X Zou J Wang Z. Early change in peripheral CD4(+) T cells associated with clinical outcomes of immunotherapy in gastrointestinal cancer. Immunotherapy (2021) 13(1):55–66. doi: 10.2217/imt-2020-0068 33086925

